# Comparing Mannitol and Hypertonic Dextrose Injections for Knee Osteoarthritis Pain and Function: A Randomised Trial

**DOI:** 10.31138/mjr.280224.cmd

**Published:** 2025-03-31

**Authors:** Nasrin Barzegar, Rezvan Ghaderpanah, Hamid Reza Farpour, Mohammad Esmaeil Ghorbani Nejad

**Affiliations:** 1 Student Research Committee, Department of Physical Medicine and Rehabilitation, Shiraz University of Medical Sciences, Shiraz, Iran;; 2 Orthopaedic and Rehabilitation Research Centre, Department of Physical Medicine and Rehabilitation, Shiraz University of Medical Sciences, Shiraz, Iran;; 3 Shiraz Geriatric Research Centre, School of Medicine, Shiraz University of Medical Sciences, Shiraz, Iran

**Keywords:** intra-articular injections, mannitol, hypertonic glucose solution, hypertonic dextrose, prolotherapy

## Abstract

**Introduction:**

Knee osteoarthritis is a chronic and age-related disease that causes joint stiffness, pain, and biomechanical changes in the joint, resulting in decreased activity and performance. Prolotherapy is one of the methods of injection therapy in the management of this disease. Drugs such as hypertonic dextrose and mannitol have been introduced as prolotherapy drugs. The aim of this study is to evaluate the efficacy of intra-articular injection of mannitol compared to prolotherapy with hypertonic dextrose in terms of pain relief and functional improvement in patients with knee osteoarthritis.

**Patients and Methods:**

A total of 48 patients with KOA were randomly divided into two groups: hypertonic dextrose (24 patients) and mannitol (26 patients). All patients received three intra-articular injections of either hypertonic dextrose or mannitol at two-week intervals. Visual Analogue Pain Scale (VAS), Oxford Knee Scale (OKS), and Western Ontario McMaster University Osteoarthritis Index (WOMAC) questionnaire scores were the outcome measures assessed before and 2, 4, and 8 weeks after the injections.

**Results:**

There were no statistically significant differences in pre-injection demographic characteristics between the two groups (p > 0.05). Results showed that VAS and OKS scores decreased over time (p < 0.001). Both interventions significantly improved the mean scores of WOMAC pain, WOMAC stiffness, WOMAC function, and WOMAC total score. There were not any serious side effects in any of the groups.

**Conclusion:**

The results showed that prolotherapy is an effective and safe treatment. Although both groups had improvements in outcome measures during follow-up up to 8 weeks after the intervention, no statistically significant difference was found between the two groups.

## INTRODUCTION

Osteoarthritis, the most common type of arthritis, is the main reason of musculoskeletal disability and pain worldwide and imposes an enormous economic burden on society and individuals. The prevalence of symptomatic knee OA in adults over 60 years of age is approximately 10% in men and 13% in women. The total number of individuals affected by symptomatic OA is expected to increase due to the aging population and the obesity epidemic.^1^

The management of osteoarthritis (OA) involves various approaches such as medications, physical therapy, occupational therapy, and surgery, with the goal of relieving pain and improving joint function.^2,3^ Despite ongoing efforts, treatments that effectively modify the progression of OA have not yet achieved sufficient efficacy for widespread approval. The American College of Rheumatology (ACR) guidelines recommend non-surgical treatments, although these methods do not address the underlying cause of OA.^4^

One of the therapies for KOA, which may be recommended, is intra- and periarticular injections into the knee. Platelet-rich plasma (PRP), hyaluronic acid, methylprednisolone, and human platelet lysate are some of the substances used for intra-articular knee injections. Hypertonic dextrose injection, also known as “prolotherapy,”^5^ has attracted much attention since it was first used to treat musculoskeletal pain in the 1950s. It is an injection-based treatment that has been used in recent years for various painful chronic musculoskeletal conditions such as tendinitis and OA.^6^

The main principle of prolotherapy is the injection of relatively small volumes (0.5-6 ml) of an irritant solution to painful ligament and tendon insertions and into the adjacent joint spaces.^5^ Although it has been used as a regenerative injection for several years, the exact mechanism is still unknown. It is known that prolotherapy targets ligament laxity, which primarily contributes to the development of osteoarthritis by activating an inflammatory cascade that attracts fibroblasts and stimulates collagen production.^5,7^ One theory suggests that the attraction of inflammatory mediators and release of growth factors is caused by the hyperosmolar dextrose solution, which acts as a cell irritant.^8^ It is believed that the strengthening of the ligaments supporting the joint restores the biomechanical function of the joint and leads to pain relief. Recently, prolotherapy injections were discovered to have disease-modifying effects in knee OA, particularly by stimulating cartilage metabolism, leading to the growth of fibrous and hyaline cartilage.^9^ Animal studies, on the other hand, have repeatedly shown that peritendinous dextrose injection results in fibroblast and vascular proliferation, dense collagen deposition, improved ligament thickness, energy absorption, and ultimate resilience.^10^

On the other hand, according to some studies, mannitol can alleviate pain and improve function by downregulating the transient receptor potential vanilloid type 1 (TRPV-1) receptor, which restores neuronal activity and leads to a return to normal innervation.^11^ Conrozier et al. discovered in a study that mannitol, a polyol known for its ability to scavenge reactive oxygen compounds (ROS), can improve the intra-articular residence time of hyaluronic acid (HA).^12^ In an animal model, mannitol has also shown anti-inflammatory activity.^13^

The positive results of the studies suggested that prolotherapy can be beneficial, but the limitations of these studies do not allow for a rigorous evaluation of efficacy. The therapeutic value, use of prolotherapy, and efficacy are controversial because the experimental and clinical evidence is insufficient to conclude.^5^ This study compared the efficacy of intra-articular injections of hypertonic dextrose versus mannitol in relieving pain, stiffness and improving function in people with knee osteoarthritis

## MATERIALS AND METHODS

### Study design, participants and sample size

This was a randomised, double-blind clinical trial. Patients referred to the Rajai and Imam Reza clinics affiliated with Shiraz University of Medical Sciences’ physical medicine and rehabilitation institutes between April and November 2020 were eligible to participate. Statistical analysis of data from similar studies was used to determine the sample size, based on a significance level of 0.5 (p<0.05), confidence interval of 95%, power of 80%, and sampling loss of 20%. Consequently, the sample size for each group was set at 25 patients.

### Selection Criteria

#### Inclusion criteria

1) Patients aged 38 to 70 years; 2) willingness to take part in the study; 3) with clinical symptoms of KOA (based on American College of Rheumatology clinical criteria^14^) for more than 3 months; 4) with a Kellgren-Lawrence Scale grade 1 or 2 (mild) on radiograph^15^ within the past 1 month; 5) a Visual Analog Scale of at least three^16^; 6) no pathologic problems around the knee, such as bursitis or cellulitis.

### Exclusion criteria

1) Systemic diseases such as diabetes, rheumatoid arthritis, haematologic diseases (coagulopathies), severe cardiovascular diseases, infections or immunodeficiency; 2) previous arthroplasty; 3) intra-articular or periarticular injection in the previous three months; 4) body mass index (BMI) greater than 40 kg/m^2^; 5) severe knee osteoarthritis as determined by radiologic imaging (grades III and IV based on Kellgren-Lawrence radiologic criteria); 6) history of trauma in the past three months; 7) active lumbosacral radiculopathy, peripheral neuropathy, or myopathy; 8) previous knee prolotherapy; 9) inflammatory or post-infectious knee osteoarthritis; 10) daily use of opioids; 11) drug allergy or intolerance; 12) failure to complete surveys; 13) any sign of redness, hotness, or effusion around the knee; 14) pregnancy.

### Randomisation and allocation

After implementing the exclusion criteria, we divided patients using computer-based software with a block randomisation protocol. Patients were randomly allocated to one of two study groups: knee-intra-articular injection of hypertonic Dextrose or injection of knee-intra-articular mannitol. Patients and investigators assessing the treatment were unaware of the treatment allocation.

### Intervention

Participation in the study was on a voluntary basis. After describing RCT, we obtained informed consent from the patients before enrolling them in the study. The possible complications of knee injections in this study, we explained the complications thoroughly. It should be noted that the investigators followed up on all issues. In all groups, patients were given a phone number to contact if they had any questions. At each follow-up, treatment complications were assessed. Each patient was in the supine position, with knee flexion of 10 to 15°. The injector examined the knee, and the skin was prepared and draped. After aspiration and verification of correct needle placement in the joint, the injection was done with the G23 needle. In group A, injection was performed with sterile disposable syringes with a mixture of 2.5 ml dextrose 50% + 2.5 ml 2% lidocaine. In group B, each patient was injected with a mixture of 1 ml mannitol 20% + 4 ml 2% lidocaine (XYLEX, manufactured by Sina Pharmaceutical). After two weeks, the injection was repeated (each patient received three sessions of intra-articular injection). Finally, all patients were instructed to use a cold compress for 5 minutes in case of pain or bruising at the injection site. We also advised them to correct their lifestyle and do appropriate exercises to lower knee pain, as instructed in the clinic. Patients could also take paracetamol tablets if they felt pain. Three intra-articular injections were given to each patient at 0, 2, and 4 weeks apart.

### Outcomes

Patients enrolled in the study were asked to complete the following forms and questionnaires based on the selection criteria:

1. Patient profile form, which includes demographic information, medical history, previous knee injections, and history of hypersensitivity to topical treatments.
2. Visual analog scale (VAS)^16^ used to assess pain intensity, with 0 representing no pain and 10 representing the most pain.
3. Western Ontario and McMaster Universities Arthritis Index (WOMAC),^17^ is a three-part index used to assess patient function. The first component is daily functional pain consisting of 5 items, the second part measures pain with various daily activities and joint dryness (2 items), and the third component measures physical function by a limp examination with 17 items. The WOMAC total score is composed of 24 components, each comprising five scales (0–4). The WOMAC score ranges from 0 to 96, with 96 indicating the worst function and a decrease in WOMAC score indicating improvement.^18^
4. The Oxford Knee Scale (OKS)^18^ consists of 12 different components with five different scales ranging from 0 to 4. The questionnaire assesses the ability to perform various activity functions and assigns a score from 0 to 48 for best performance; thus, an increase in OKS score indicates improved performance.^17^

### Statistical Analysis

The results of this study were analysed using SPSS version 22 software, which included descriptive (mean and standard deviation) and analytical tests (Kolmogorov-Smirnov and t-test).

## RESULTS

After excluding four patients, we randomly divided the 48 remaining participants into two groups: Mannitol (group A) and hypertonic dextrose (group B) in equal proportions and studied them over a 12-month period. Two individuals refused to participate in this study (both in group B), and two others were traumatised and excluded from the study (both in group A). **Table 1** summarises all demographic information on these two groups (without statistical significance). The mean score of the OKS questionnaire (an indicator of pain and function) before treatment was 22.58 and 23.29 for the mannitol and HD groups, respectively (No significant difference, p=0.81). At two, four, and eight weeks post-injection follow-up, the improvements in pain and function were not statistically significant. During follow-up, significant improvements gradually occurred in both groups (statistically significant, p < 0.001).

**Table 1.** Characteristics of the patients in mannitol group and dextrose group.

	**Mannitol group (Group A)**	**Hypertonic dextrose group (Group B)**	**P value**
Sex			

Male, n (%)	7 (29.2)	8 (33.3)	0.75
Female, n (%)	17 (70.8)	16 (66.6)	

Age, mean±SD (year)	51.79±8.61	53.54±9.72	0.54

BMI, mean±SD (kg/m^2^)	27.77±2.49	28.04±2.78	0.84

BMI: body mass index; SD, standard deviation.

**Figure 2.**
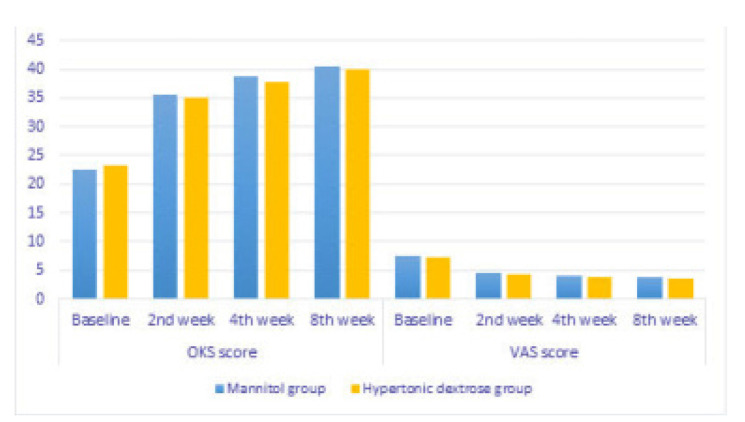
Comparison of OKS and VAS score between groups.

The VAS questionnaire scores were statistically the same as the OKS and showed no significant difference before or two, four, or eight weeks after injection. However, the VAS scores showed that pain decreased significantly over time in both groups (p < 0.001). (**Table 2**, **Figure 2**).

**Table 2.** Comparison of OKS score and VAS score in mannitol and hypertonic dextrose group.

		**Mannitol group (mean±SD)**	**Hypertonic dextrose group (mean±SD)**	**P value (between groups)**	**P value (within groups)**
OKS score	Baseline	22.58±4.10	23.29±2.94	0.819	< 0.001
	2^nd^ week	35.70±3.23	35.16±3.87	0.383	
	4^th^ week	38.87±2.78	37.83±3.77	0.135	
	8^th^ week	40.58±3.38	40.00±3.56	0.554	

VAS score	Baseline	7.40±0.59	7.37±0.64	0.718	< 0.001
	2^nd^ week	4.45±0.80	4.29±0.95	0.621	
	4^th^ week	4.04±0.78	3.83±0.91	0.484	
	8^th^ week	3.81±0.79	3.66±0.86	0.640	

PTH: parathyroid hormone; P1NP: type 1 collagen propeptide; CTX: C-terminal telopeptide.

The pre-intervention WOMAC total score was 49.58 (mannitol) and 51.58 (HD), with no significant difference (p=0.21). Neither the WOMAC total score nor the subscale scores (pain, stiffness, and function) had statistically significant differences between the two groups at any time point; however, all subscales improved significantly over time in both groups (p < 0.001).

## DISCUSSION

Our results show that intra-articular injection of mannitol and hypertonic dextrose (as local treatments) has a significant effect on functional improvement and pain reduction in patients with knee osteoarthritis (p < 0.05). OKS and VAS scores improved by approximately 35% in each group relative to baseline status. The WOMAC total scores showed an improvement of approximately 40% in each group, and the function subscale showed the best improvement among all subscales with an improvement of 28%. However, there was no significant difference in VAS, OKS, and WOMAC scores between these two groups at the second, fourth, or eighth week after treatment with hypertonic dextrose. Although systemic therapy is significantly limited in patients with KOA, especially in the elderly, significant adverse effects have rarely been reported for these local treatments, and no adverse effects occurred in our study.

The results of our study are consistent with some other studies^19–22^ confirming the clinical efficacy of hypertonic dextrose as a safe and cost-effective solution that is easy to use in clinical practice. Moreover, the injection can be performed in an outpatient setting. According to a study by Rabago et al. performed on 98 patients with knee osteoarthritis, dextrose injection was the best intervention compared to two other groups (saline and dextrose group). Dextrose injection was able to improve WOMAC scores by 24% compared to baseline. The results also showed that prolotherapy of the knee can provide sustained improvement in quality of life, pain, and stiffness can be considered a preferred method in patients who do not respond to conservative treatment.^23^ A systematic review and meta-analysis by WS Sit et al. included 258 patients with moderate pain and showed that prolotherapy was superior to exercise. The WOMAC composite, functional, and pain scales were improved by a standardised mean difference (SMD) of 0.81, 0.78, and 0.62, respectively (p < 0.001).^21^ Based on a recent study by Babaeian et al. conducted on 54 patients with KOA who received HD as prolotherapy, all VAS, OKS, and WOMAC scores improved significantly compared with baseline.^20^

**Figure 1.**
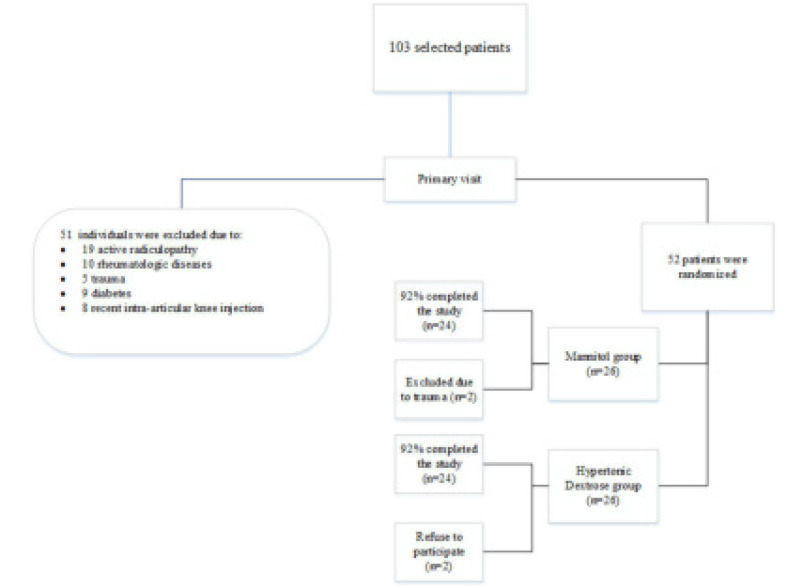
Study consort flowchart.

**Table 3.** Comparison of WOMAC score in mannitol and hypertonic group.

		**Mannitol group (mean±SD)**	**Hypertonic dextrose group (mean±SD)**	**P value (between groups)**	**P value (within groups)**
WOMAC score	Baseline	49.58±5.67	51.58±5.04	0.215	< 0.001
	2^nd^ week	22.20±8.39	22.16±7.37	0.901	
	4^th^ week	15.16±5.70	16.66±4.56	0.321	
	8^th^ week	11.16±7.01	12.25±5.43	0.553	

Pain score	Baseline	10.95±1.92	11.62±2.06	0.285	< 0.001
	2^nd^ week	5.08±2.30	5.04±2.45	0.958	
	4^th^ week	3.54±1.71	3.62±1.61	0.941	
	8^th^ week	2.20±1.97	2.66±1.71	0.390	

Stiffness score	Baseline	3.37±1.34	3.70±1.19	0.195	< 0.001
	2^nd^ week	1.37±1.09	1.54±1.02	0.563	
	4^th^ week	1.04±.095	1.37±0.92	0.202	
	8^th^ week	0.70±0.95	1.16±0.96	0.102	

Function score	Baseline	35.25±3.50	36.25±3.26	0.311	< 0.001
	2^nd^ week	15.33±5.79	15.58±4.88	0.794	
	4^th^ week	10.58±4.32	11.66±2.97	0.317	
	8^th^ week	8.25±5.28	8.41±3.68	0.605	

WOMAC: Western Ontario and McMaster Universities Arthritis Index; SD, standard deviation.

On the other hand, mannitol showed promising results in reducing pain and improving function in several studies. According to a study that investigated the safety and efficacy of a hyaluronic acid-mannitol mixture over a six-month period, drug use and joint function improved significantly.^24^ The results of another study with a follow-up period of 24 months also showed that mannitol significantly improved WOMAC scores in all subscales.^25^ In patients with KOA, Conrozier et al. investigated the safety and efficacy of a new intra-articular viscosupplement of medium molecular weight hyaluronic acid (HA) in combination with a high concentration of mannitol compared with a conventional high dose of HA. This study found that therapy with this mixture effectively reduced symptoms of knee OA and improved joint function over a six-month period while being as safe as the standard viscosupplement HA.^26^ Furthermore, in a study measuring pain scores after administration of topical mannitol, Bertrand et al. discovered that mannitol may have an analgesic effect by downregulating TRPV1 receptors. TRPV1 is involved in the perception and regulation of pain.^11^ In another study, Hashemi et al. compared the short-term benefits of prolotherapy with dextrose for functional improvement and pain relief in knee osteoarthritis with intraarticular hyaluronic acid injections. According to the results of this study, intra-articular injections of 25% dextrose prolotherapy may be as beneficial as hyaluronic acid injections in relieving knee pain caused by OA.^27^ In a recent study, evaluating 936 individuals involved in fourteen separate studies, dextrose exhibited substantial pain relief when contrasted with saline, exercise, intra-articular HA, PRP, and alternative interventions. Noteworthy findings revealed that dextrose consistently outperformed saline and exercise in alleviating pain intensity and enhancing functional outcomes, as corroborated by WOMAC, VAS, and other evaluative measures. However, when juxtaposed with HA, dextrose yielded varied outcomes in terms of pain reduction and functional improvement.^28^

## LIMITATION AND FUTURE STUDIES

Our study has certain limitations: the lack of a control group is the first limitation of our study. Second, our study includes a short follow-up period in which no adverse events such as drug intolerance or rare injection-related sequelae were observed. In addition, generalisability may be limited by a large number of exclusion criteria and a relative scarcity of participants with high baseline WOMAC scores. It is suggested that future studies examine a larger number of participants over a longer period of time. We also recommend the use of the EuroQol-5D to measure the cost-effectiveness of this intervention,^29^ and objective assessments such as magnetic resonance imaging and ultrasound for more accurate evaluation.^6^

Looking ahead, future studies on Prolotherapy can focus on finding the right amount of treatment and how often sessions should occur for different types and stages of osteoarthritis (OA). It’s also important to explore how effective prolotherapy is for joints besides the knee, like the hip, shoulder, or spine. Studying prolotherapy over the long term and comparing it with new treatments will help us understand how well it works over time and how safe and effective it is compared to other options for managing OA.

## CONCLUSION

Since systemic treatments for KOA are severely limited, our study has provided substantial insights into local treatments for this condition. Despite its limitations, this study suggests that three intra-articular injections of mannitol or dextrose can significantly relieve pain and improve function in patients with knee osteoarthritis. Because we did not find significant differences between these two methods, future studies should focus on comparing these two approaches individually in placebo-controlled groups with larger populations.

## AUTHOR’S CONTRIBUTIONS

Nasrin Barzegar: Conceptualisation, Methodology, Data curation, Review & Editing draft; Rezvan Ghaderpanah: Data curation, Writing- Original draft preparation, Formal analysis; Hamid Reza Farpour: Visualisation, Investigation, Conceptualisation, Methodology, Review & Editing manuscript, and Project administration; Mohammad Esmaeil Ghorbani Nejad: Formal analysis, Software, Writing- Original draft. All authors commented on the previous versions of the manuscript and revised it. All authors read and approved the final manuscript.

## CONFLICT OF INTEREST

The authors declare no conflict of interest.

## FINANCIAL SUPPORT

This study is a part of the thesis of Nasrin Barzegar (Grant no: 19583), supported by Shiraz University of Medical Sciences.

## ETHICAL CONSIDERATIONS

The manuscript has been approved by all authors and has never been published or under the consideration for publication elsewhere. We confirm that all figures and tables are original and created by authors. We guarantee that all authors listed on the title page have read the manuscript and attest to the validity and legitimacy of the data. The study was conducted in accordance with the ethical standards of the Declaration of Helsinki and Good Clinical Practice. It was also approved by the Ethics Committee of Shiraz College of Medical Sciences (ethics number: IR.SUMS. MED. REC.1398.314) and registered with clinical trial registration number: IRCT20190912044756N1, registration date: 2020-08-24.

## DATA AVAILABILITY STATEMENT

The data supporting this study’s findings are available from the corresponding author upon reasonable request.
